# Novel, Objective, Multivariate Biomarkers Composed of Plasma Amino Acid Profiles for the Diagnosis and Assessment of Inflammatory Bowel Disease

**DOI:** 10.1371/journal.pone.0031131

**Published:** 2012-01-31

**Authors:** Tadakazu Hisamatsu, Susumu Okamoto, Masaki Hashimoto, Takahiko Muramatsu, Ayatoshi Andou, Michihide Uo, Mina T. Kitazume, Katsuyoshi Matsuoka, Tomoharu Yajima, Nagamu Inoue, Takanori Kanai, Haruhiko Ogata, Yasushi Iwao, Minoru Yamakado, Ryosei Sakai, Nobukazu Ono, Toshihiko Ando, Manabu Suzuki, Toshifumi Hibi

**Affiliations:** 1 Department of Internal Medicine, School of Medicine, Keio University, Tokyo, Japan; 2 Center for Diagnostic and Therapeutic Endoscopy, Keio University, Tokyo, Japan; 3 Center for Multiphasic Health Testing and Services, Mitsui Memorial Hospital, Tokyo, Japan; 4 Institute of Life Sciences and Pharmaceutical Research Laboratories, Ajinomoto Co. Inc., Kawasaki, Japan; Ulm University, Germany

## Abstract

**Background:**

Inflammatory bowel disease (IBD) is a chronic intestinal disorder that is associated with a limited number of clinical biomarkers. In order to facilitate the diagnosis of IBD and assess its disease activity, we investigated the potential of novel multivariate indexes using statistical modeling of plasma amino acid concentrations (aminogram).

**Methodology and Principal Findings:**

We measured fasting plasma aminograms in 387 IBD patients (Crohn's disease (CD), n = 165; ulcerative colitis (UC), n = 222) and 210 healthy controls. Based on Fisher linear classifiers, multivariate indexes were developed from the aminogram in discovery samples (CD, n = 102; UC, n = 102; age and sex-matched healthy controls, n = 102) and internally validated. The indexes were used to discriminate between CD or UC patients and healthy controls, as well as between patients with active disease and those in remission. We assessed index performances using the area under the curve of the receiver operating characteristic (ROC AUC). We observed significant alterations to the plasma aminogram, including histidine and tryptophan. The multivariate indexes established from plasma aminograms were able to distinguish CD or UC patients from healthy controls with ROC AUCs of 0.940 (95% confidence interval (CI): 0.898–0.983) and 0.894 (95%CI: 0.853–0.935), respectively in validation samples (CD, n = 63; UC, n = 120; healthy controls, n = 108). In addition, other indexes appeared to be a measure of disease activity. These indexes distinguished active CD or UC patients from each remission patients with ROC AUCs of 0.894 (95%CI: 0.853–0.935) and 0.849 (95%CI: 0.770–0.928), and correlated with clinical disease activity indexes for CD (r_s_ = 0.592, 95%CI: 0.385–0.742, p<0.001) or UC (r_s_ = 0.598, 95%CI: 0.452–0.713, p<0.001), respectively.

**Conclusions and Significance:**

In this study, we demonstrated that established multivariate indexes composed of plasma amino acid profiles can serve as novel, non-invasive, objective biomarkers for the diagnosis and monitoring of IBD, providing us with new insights into the pathophysiology of the disease.

## Introduction

Inflammatory bowel disease (IBD) is a chronic intestinal disorder comprising two major types, Crohn's disease (CD) and ulcerative colitis (UC) [1], [2]. Despite intensive research, the etiology of IBD remains unknown, although it is considered to be a multi-factorial disease determined by genetic backgrounds, environmental factors and immunological disorders.

Importantly, the number of patients with IBD and colorectal cancers in Asia has increased remarkably during the past decade. One of the reasons for this change is thought to be the move towards a more Westernized diet. Dietary habits are recognized to be an important modifiable environmental factor influencing the risk of these diseases. Most physicians believe in the role of diet and nutritional metabolism in IBD pathogenesis, however, clinical and basic research has not adequately addressed these issues.

The human body is a highly organized metabolic network of systems that regulates individual homeostasis, but it is often difficult to objectively assess. Analysis of the metabolomic condition instead is therefore of use in determining health status, as disturbances of metabolic homeostasis are known to be related to the pathogenesis of metabolic syndromes, chronic inflammatory disorders, and cancers. Post-genomic technologies, in particular metabolomics, provide new opportunities to study metabolic effects in relation to disease. Metabolomics is a rapidly evolving field that comprehensively measures metabolites, ideally in a biological fluid, and changes in metabolic profiles are a potential source of biomarkers.

Overall, 20% of the human body is composed of amino acids (AAs) and their metabolites, which play important roles as both basic substrates and regulators in many metabolic pathways [3], [4]. Specific abnormalities in plasma AA concentrations have been reported in the context of various diseases, such as Fischer's ratio in fibrotic liver disease [5], [6], [7], [8]. Plasma AA profiling is also a potential screening tool for non-small cell lung carcinoma (NSCLC) [9].

Previously, we demonstrated that aminograms and the generation of a multivariate index using “AminoIndex_™_ technology” (MIAI) have the potential for diagnostic use, disease activity monitoring, and the assessment of pathophysiological conditions [10]. Here, we introduced the concept of metabolomics to analyse AA metabolism in IBD patients, and found that AA metabolism was disturbed in IBD patients, particularly those with active disease. AA profiles may reflect the nutritional condition of individuals, disease activity, and differences in pathogenesis between CD and UC. Finally, we established the novel clinical parameter, the MIAI which discriminated between CD and UC, and also reflected disease activity. We demonstrate for the first time that disturbances of AA metabolism are related to the pathophysiological state of IBD and that the MIAI is a novel, non-invasive, diagnostic and monitoring marker for IBD.

## Methods

### Patients

IBD patients (n = 387) were recruited between February 27, 2005, and March 7, 2008, at the Keio University Hospital, Tokyo, Japan. Healthy controls (HCs; n = 210) were recruited between December 1, 2005, and April 1, 2006, from the Center for Multiphasic Health Testing and Services, Mitsui Memorial Hospital, Tokyo, Japan. Patients and HCs were divided into discovery and validation sets. For the discovery screening, plasma samples were obtained from CD patients (n = 102), UC patients (n = 102) and age and sex-matched HCs (n = 102). For the validation test, plasma samples were obtained from CD patients (n = 63), UC patients (n = 120) and HCs (n = 108).

Patient and HC characteristics are shown in Table 1. For continuous monitoring, IBD patients (n = 22) were recruited between May 30, 2005, and February 18, 2010, at Keio University Hospital. This study was conducted in accordance with the Declaration of Helsinki, and the protocol was approved by the ethics committees of the Keio University School of Medicine and the Mitsui Memorial Hospital. Signed informed consent forms were obtained from all patients and all data were analyzed anonymously throughout the study. The diagnosis of UC and CD was based on established clinical, radiographic, endoscopic, and histopathologic criteria. Patient characteristics were determined from medical records, questionnaires, and interviews. Disease activity was assessed by the Crohn's disease activity index (CDAI) [11] and the *Lichtiger* Clinical Activity Index (CAI) [12]. Active disease was defined as CDAI≥150 for patients with CD and CAI≥5 for patients with UC. Remission was defined as CDAI<150 for patients with CD and CAI<5 for patients with UC.

**Table 1:** Patient characteristics.

		CD	UC	HC
Discovery Set	N	102	102	102
	Age, years	36.1±9.9	35.9±9.4	36.4±8.6
	Female, n (%)	32 (31)	32 (31)	32 (31)
	Mean disease duration, years	11.0±7.4	7.8±6.7	–
	Mean age at diagnosis, years	25.1±7.7	28.1±8.5	–
	Active disease	29	38	–
	CD characteristics			
	Disease location			
	Small bowel	31	–	–
	Colon	9	–	–
	Both	59	–	–
	Others	3	–	–
	Behavior (Montreal classification)			
	B1: non-stricturing, non-penetrating	26	–	–
	B2: stricturing	42	–	–
	B3: penetrating	34	–	–
	p: perianal disease modifier	40	–	–
	UC characteristics			
	Disease location			
	Proctitis	–	12	–
	Left sided colitis	–	43	–
	Entire colitis	–	47	–
	Treatment			
	Enteral nutrition	44	1	–
	Steroids	12	37	–
	Salicylates	91	98	–
	Immunosuppressors	56	25	–
	Infliximab	14	0	–
	Biomarker levels	Median (interquartile range)		
	Albumin (mg/dl)	4.2 (3.8–4.4)	4.5 (4.1–4.6)	4.6 (4.4–4.8)
	C-reactive Protein (mg/dl)	0.22 (0.03–0.77)	0.19 (0.03–0.25)	0.02 (0.01–0.04)
	Hemoglobin (g/dl)	12.8 (11.5–13.9)	13.9 (13.3–14.8)	15.2 (13.6–15.7)
Validation Set	n	63	120	108
	Age, years	32.3±11.2	42.6±16.2	42.4±7.9
	Female, n (%)	20 (33)	40 (33)	34 (32)

Plus-minus values are means±standard deviation.

### Plasma AA analysis

Plasma samples for AA analysis were obtained using EDTA as an anticoagulant and stored at −80°C until analysis. Frozen plasma samples were briefly thawed, mixed with 5-sulfosalicylic acid (final concentration, 2.3%), then centrifuged for 10 min at 11,000× *g* at 4°C to remove precipitated proteins. Measurement of AA concentrations was performed using an automatic AA analyzer (L-8500; Hitachi High-Technologies Corporation, Tokyo, Japan). Briefly, AAs separated by cation-exchange chromatography were detected spectrophotometrically after a post-column reaction with ninhydrin reagent. We confirmed that the fasting plasma aminograms remained constant for individuals in this protocol.

### Statistical analysis

For comparisons between study groups, we used the Mann-Whitney U test with Bonferroni correction. The Spearman correlation was used to test associations of CDAI, CAI and serum C-reactive protein (CRP) with the MIAI. These univariate statistical analyses were performed using Prism software, version 5.0.1 (Graph Pad Software, San Diego, CA). Correlation scatterplot analyses were carried out using JMP software, version 6.0.3 (SAS Institute Inc., Cary, NC). Multiple discriminant analyses were performed in the discovery set, then validated in the validation set with MATLAB software, version 7.6.0 (Mathworks, Natick, MA). Model performances were assessed using the area under the ROC curve (ROC AUC) as a measure of the validity of the MIAI. Details are given in Methods S1.

## Results

### Plasma His and Trp concentrations in IBD patients

Analysis of serum protein levels showed that serum albumin, but not total protein level, was decreased in IBD patients. Interestingly, the concentrations of several plasma AAs (e.g. histidine (His) and tryptophan (Trp)) were significantly decreased, while the levels of others were maintained in IBD patients (Table 2). Therefore, the observed changes in the concentrations of plasma AAs suggested that there is a metabolic alteration that occurs in patients with IBD.

**Table 2:** Comparison of plasma amino acid (AA) concentrations in healthy control subjects, Crohn's disease and ulcerative colitis patients.

	HC	CD	UC	p
Albumin (g/dl)	4.6±0.2	4.1±0.5^2^	4.3±0.5^2^	<0.001^1^
Total Protein (g/dl)	7.2±0.3	7.3±0.7	7.4±0.5^2^	<0.001^1^
EAA				
Valine	219 (190–241)	195 (161–216)^4^	191 (166–221)^4^	<0.001^3^
Leucine	118 (98–135)	102 (84–119)^4^	101 (85–117)^4^	<0.001^3^
Isoleucine	61 (49–68)	57 (51–65)	54 (44–63)^4^	0.028^3^
Threonine	121 (108–134)	117 (97–146)	110 (94–130)^4^	0.036^3^
Lysine	192 (172–208)	187 (163–206)	174 (148–197)^4^	0.005^3^
Methionine	27 (24–29)	25 (21–29)^4^	23 (20–26)^4^	<0.001^3^
Histidine	83 (77–89)	72 (66–79)^4^	72 (66–81)^4^	<0.001^3^
Tryptophan	49 (42–56)	45 (38–52)^4^	48 (40–55)	0.05^3^
Phenylalanine	58 (51–63)	52 (46–61)^4^	54 (47–58)^4^	<0.001^3^
NEAA				
Glutamic acid	32 (25–42)	39 (30–51)	39 (29–50)	<0.001^3^
Asparagine	46 (41–51)	41 (37–48)^4^	40 (35–46)^4^	<0.001^3^
Serine	114 (98–129)	107 (97–122)	110 (85–124)	0.137^3^
Glutamine	565 (525–613)	544 (494–592)	550 (474–590)^4^	0.019^3^
Glycine	231 (205–264)	241 (205–286)	218 (185–251)^4^	0.002^3^
Proline	122 (105–144)	145 (114–181)^4^	125 (102–166)	0.001^3^
Tyrosine	61 (55–68)	52 (44–60)^4^	53 (46–60)^4^	<0.001^3^
Arginine	91 (78–103)	89 (74–104)	87 (72–96)	0.254^3^
Alanine	319 (265–362)	322 (261–379)	308 (271–356)	0.887^3^
Other				
Citrulline	31 (26–35)	28 (23–35)	28 (23–32)^4^	0.003^3^
Taurine	49 (44–56)	73 (58–99)^4^	68 (53–86)^4^	<0.001^3^
Ornithine	57 (51–68)	57 (48–67)	50 (44–58)^4^	<0.001^3^
Sum of EAAs	932 (845–1019)	856 (761–963)^4^	830 (745–915)^4^	<0.001^3^
Sum of NEAAs	1615 (1490–1714)	1463 (1104–1713)^4^	1562 (1395–1688)	0.002^3^
EAA/NEAA	0.58 (0.54–0.63)	0.58 (0.52–0.71)	0.55 (0.50–0.58)^4^	<0.001^3^

Serum albumine and total protein concentration are presented as mean±standard deviation. Plasma amino acid concentrations are presented as median (interquartile range).

1 One-way analysis of variance test.

2 Significantly different from control subjects, p<0.01 (by Dunnett's test).

3 Kruskal Wallis Test.

4 Significantly different from control subjects, p<0.01 (by Kruskal-Wallis after adjustment for CD and UC vs. HC by Dunn's test).

**Figure 1:**
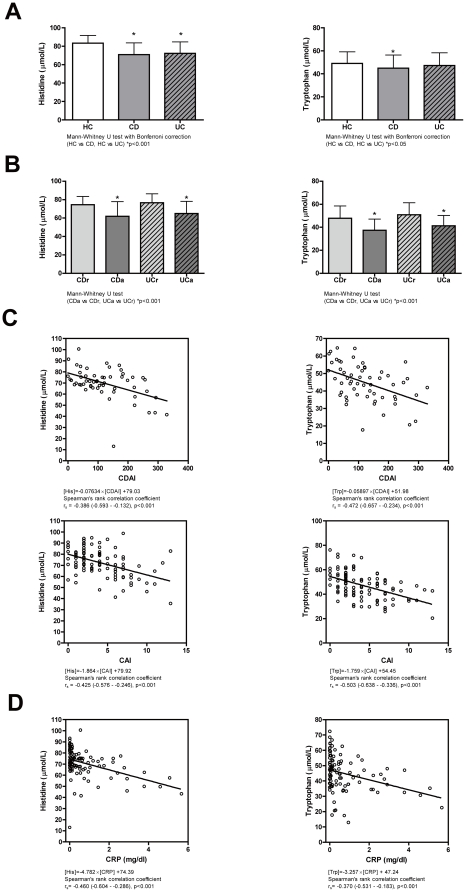
Plasma His and Trp concentrations in IBD patients. (**A**) Plasma His concentrations are significantly lower in CD (grey bars) and UC (striped bars) patients than in HCs (white bars). Plasma Trp concentrations are significantly lower in CD patients than in HCs. The two-tailed p-values are based on the Mann-Whitney U test with the Bonferroni correction. (**B**) Plasma His and Trp concentrations are significantly lower in patients with active disease (CDa, dark grey bars, and UCa, dark striped bars) than in patients in remission (CDr, light grey bars, and UCr, light striped bars), respectively. The two-tailed p-values are based on the Mann-Whitney U test. (**C**) Significant inverse correlations between plasma His and Trp concentrations and disease activities (CDAI for CD and CAI for UC). (**D**) Significant inverse correlations between plasma His and Trp concentrations and serum CRP concentrations in CD patients. The relationships in panels C and D are shown along with their Spearman's rank correlation coefficients (r_s_). Error bars show standard deviations.

As shown in Figure 1A and Table 2, plasma His concentrations were significantly decreased in both CD and UC patients compared with HCs (mean plasma His concentrations, 72.3 µM in CD patients and 71.0 µM in UC patients vs. 83.3 µM in HCs, p<0.001). Furthermore, the plasma His concentration showed significant decreases in patients with active disease (Figure 1B). Specifically, the mean plasma His concentration was 62.0 µM in CD patients with active disease (CDa) compared with 74.6 µM in CD patients in remission (CDr) (p<0.001). Similarly, the plasma His concentration was 65.0 µM in UCa patients compared with 76.6 µM in UCr patients (p<0.001). The plasma His concentration showed an inverse correlation with the CDAI in CD patients (Spearman's rank correlation coefficient r_s_ = −0.386, p<0.001) and the CAI in UC patients (r_s_ = −0.425, p<0.001) (Figure 1C). Most importantly, the plasma His concentration showed a highly inverse correlation with serum CRP levels in CD patients (r_s_ = −0.460, p<0.001) (Figure 1D and Table 3). Plasma Trp concentrations were also significantly decreased in IBD patients and inversely correlated with disease activity and serum CRP level.

**Table 3:** Spearman's rank correlation coefficients (r_s_) for plasma amino acid concentrations.

	CRP			
	CD (102)		UC (95)	
	r_s_	p	r_s_	p
Histidine	−0.46	<0.0001	−0.29	0.0041
Tryptophan	−0.37	0.0001	−0.27	0.0093
Valine	−0.08	NS	−0.06	NS
Leucine	0.02	NS	−0.02	NS
Isoleucine	−0.07	NS	−0.02	NS
Methionine	−0.25	0.0112	−0.08	NS
Phenylalanine	0.01	NS	0.05	NS
Threonine	−0.08	NS	−0.34	0.0007
Lysine	−0.17	NS	−0.01	NS
Tyrosine	0.11	NS	−0.08	NS
Serine	−0.03	NS	−0.17	NS
Asparagine	−0.24	0.0161	−0.25	0.0163
Glutamic Acid	0.29	0.0028	0.15	NS
Glutamine	−0.13	NS	−0.13	NS
Proline	−0.04	NS	−0.19	NS
Glycine	−0.07	NS	−0.22	0.0353
Alanine	−0.21	0.0327	−0.14	NS
Cystine	0.06	NS	0.05	NS
Arginine	−0.01	NS	−0.13	NS
Ornithine	0.11	NS	0.04	NS
Citrulline	−0.11	NS	−0.08	NS
Taurine	0.08	NS	−0.15	NS

NS, not significant.

### Multivariate-correlation analysis reveals alteration of AA metabolism in IBD patients

To analyze AA metabolomics in IBD patients, we measured 22 fasting plasma AA concentrations, then carried out multivariate-correlation analyses of these in IBD patients (UC: blue; CD: red) and HCs (green) (Figure S1). The scatter plots for two plasma AAs showed various patterns, and we observed a general imbalance of branched-chain AAs (BCAAs) in IBD patients (Figure S2A, B).

### MIAI for clinical diagnosis of IBD

**Figure 2:**
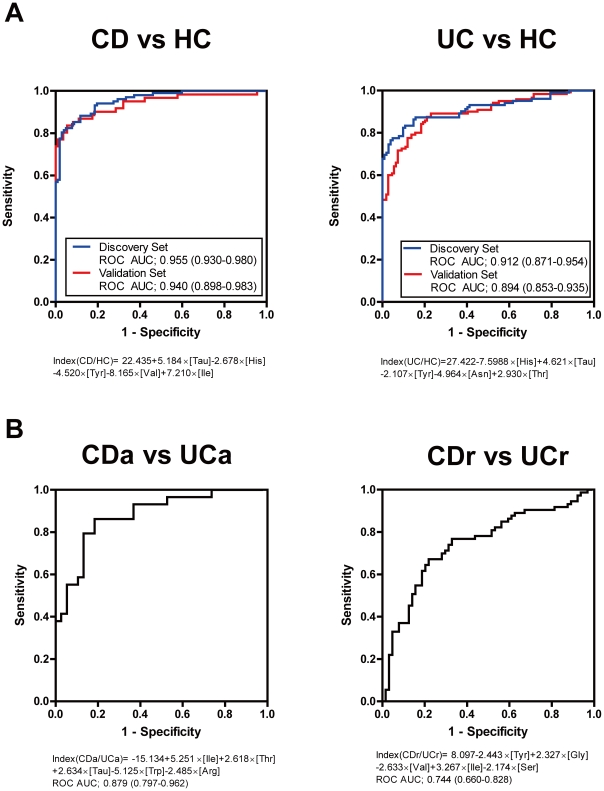
MIAI for clinical diagnosis of IBD. (**A**) ROCs of the MIAI for discriminating CD patients from HCs (Index (CD/HC) = 22.435+5.184×[Tau]−2.678×[His]−4.520×[Tyr]−8.165×[Val]+7.210×[Ile]) and UC patients from HCs (Index (UC/HC) = 27.422−7.5988×[His]+4.621×[Tau]−2.107×[Tyr]−4.964×[Asn]+2.930×[Thr]). (**B**) ROCs of the MIAI for discriminating CD and UC patients in discoveryset (Index (CDa/UCa) = −15.134+5.251×[Ile]+2.618×[Thr]+2.634×[Tau]−5.125×[Trp]−2.485×[Arg], ROC AUC = 0.879; Index (CDr/UCr) = 8.097−2.443×[Tyr]+2.327×[Gly]−2.633×[Val]+3.267×[Ile]−2.174×[Ser], ROC AUC = 0.744). CDa, active CD; CDr, remission CD; UCa, active UC; UCr, remission UC.

Although we found that AA metabolism is altered in IBD patients, it is difficult to establish a biomarker for clinical use based on these measurements. To overcome this, we tried to assess the AA metabolic condition of individuals by multivariate discriminant analysis of the plasma aminogram in the discovery set. The MIAI established from the plasma aminograms discriminated CD and UC patients from HCs with high specificity and sensitivity in the discovery set: (Index (CD/HC) = 22.435+5.184×[Tau]−2.678×[His]−4.520×[Tyr]−8.165×[Val]+7.210×[Ile], ROC AUC = 0.955; Index (UC/HC) = 27.422−7.5988×[His]+4.621×[Tau]−2.107×[Tyr]−4.964×[Asn]+2.930×[Thr], ROC AUC = 0.912) (Figure 2A and Table S1). These were then validated in additional IBD patients (ROC AUC of 0.940 for Index (CD/HC) and of 0.894 for Index (UC/HC), respectively) (Figure 2A). Importantly, the AA components between Index (CD/HC) and Index (UC/HC) differed, suggesting that AA metabolism differs between the two diseases. Therefore, the MIAI may reflect not only the inflammatory condition, but also the disease-specific AA metabolism of IBD patients.

According to these observations, we hypothesized that the MIAI is also useful for discrimination between CD and UC patients. As shown in Figure 2B, the MIAI could discriminate between CDa and UCa patients (Index (CDa/UCa) = −15.134+5.251×[Ile]+2.618×[Thr]+2.634×[Tau]−5.125×[Trp]−2.485×[Ar], ROC AUC = 0.879). By contrast, the MIAI showed less sensitivity and specificity for discrimination between CDr and UCr patients (Index (CDr/UCr) = 8.097−2.443×[Tyr]+2.327×[Gly]−2.633×[Val]+3.267×[Ile]−2.174×[Ser], ROC AUC = 0.744) (Figure 2B). These results indicate that the differences in metabolic conditions between UC and CD are more defined in active diseases, suggesting that an MIAI based on plasma AA profiling has the potential for diagnostic use.

### MIAI for assessing IBD disease activity

**Figure 3:**
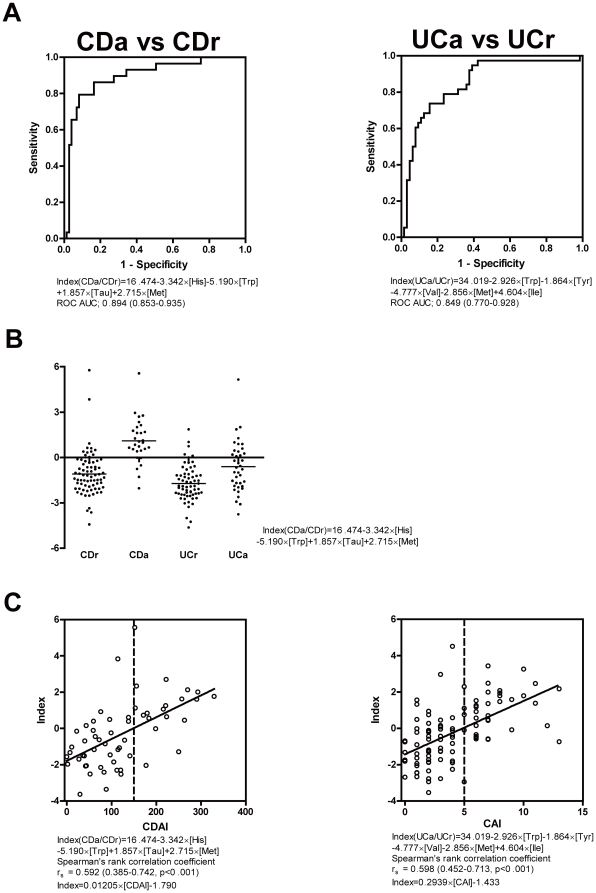
MIAI for assessing IBD disease activity. (**A**) ROCs of the MIAI for discriminating CD and UC patients with active disease and those in remission (Index (CDa/CDr) = 16.474−3.342×[His]−5.190×[Trp]+1.857×[Tau]+2.715×[Met], ROC AUC = 0.894; Index (UCa/UCr) = 34.019−2.926×[Trp]−1.864×[Tyr]−4.777×[Val]−2.856×[Met]+4.604×[Ile], ROC AUC = 0.849). (**B**) Index (CDa/CDr) cannot discriminate between active disease and remission in UC patients. (**C**) Correlations between the MIAI and disease activity indexes. Index (CDa/CDr) is correlated with the CDAI (Index = 0.01205×[CDAI]−1.790, r_s_ = 0.592, p<0.001) and Index (UCa/UCr) is correlated with the CAI (Index = 0.2939×[CAI]−1.433, r_s_ = 0.598, p<0.001).

Next, we investigated the ability of the MIAI to assess disease activity. The MIAI could discriminate between patients with active disease and those in remission among both CD and UC patients (CDa vs. CDr: Index (CDa/CDr) = 16.474−3.342×[His]−5.190×[Trp]+1.857×[Tau]+2.715×[Met], ROC AUC = 0.894; UCa vs. UCr: Index (UCa/UCr) = 34.019−2.926×[Trp]−1.864×[Tyr]−4.777×[Val]−2.856×[Met]+4.604×[Ile], ROC AUC = 0.849) (Figure 3A). As shown in Figure 3B, Index (CDa/CDr) was unable to identify the activity of UC, indicating that this index specifically identified the activity of CD. Furthermore, Index (CDa/CDr) and Index (UCa/UCr) showed a positive correlation with the CDAI (r_s_ = 0.592, p<0.001) and CAI (r_s_ = 0.598, p<0.001), respectively (Figure 3C).

### MIAI for monitoring IBD patients

**Figure 4:**
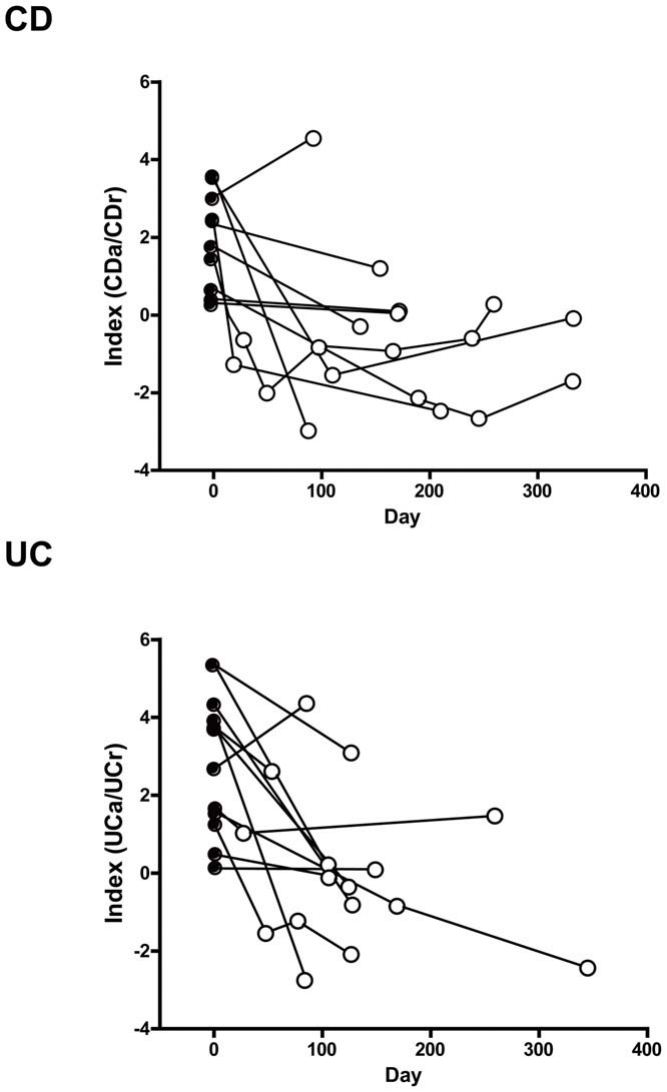
MIAI for monitoring IBD patients. Monitoring of some individual patients was performed prospectively. Upper panel shows the alterations in Index (CDa/CDr) in ten patients with active CD (CDAI≥150; closed circles). Remission was defined as CDAI<150 (open circles). Lower panel shows the alterations in Index (UCa/UCr) in 12 patients with active UC (CAI≥5; closed circles). Remission was defined as CAI<5 (open circles).

Among the biggest expectations for clinical markers are their ability to objectively monitor disease activity, their use in clinical management issues, such as when to start induction therapy and how long to continue maintenance therapy, and their ability to predict prognosis, including the risk of surgery. Regarding these issues, we continuously monitored Index (CDa/CDr) and Index (UCa/UCr) to prospectively assess disease activity in several CDa and UCa patients. As shown in Figure 4, the MIAIs were reduced by induction therapy and correlated with clinical activity. These data suggest that the MIAI may have potential as a clinical marker that can monitor disease activity in individual patients for continued observation.

## Discussion

In the present study, we have established the potential of the MIAI for clinical use based on our analyses of the plasma concentrations of 22 AAs and their metabolites in IBD patients.

Initially, we compared plasma aminograms of IBD patients with those of HCs and observed several differences, which may reflect nutritional condition, inflammation, and disease specific pathogenesis. In particular, the aminograms indicated that plasma His and Trp were significantly decreased in IBD patients with active disease. Furthermore, plasma His and Trp concentrations showed inverse correlations with disease activity indexes and serum CRP levels. These observations suggest that the decreased plasma His and Trp in IBD patients may reflect the chronic inflammatory condition, and that supplementation with His and Trp may be a novel therapeutic strategy for IBD. Trp is metabolized into kynurenine by the catabolic enzyme, indoleamine 2,3-dioxygenase (IDO), and Trp metabolism has recently been highlighted as an immunological regulator [13], [14], [15]. It is also reported that plasma kynurenine concentrations and IDO transcription levels are altered in inflamed tissue of IBD patients [16], [17].

Consistent with our observations, plasma His concentrations are decreased in several chronic inflammatory disorders such as rheumatoid arthritis and chronic kidney disease [18], [19], and are inversely correlated with the erythrocyte segmentation ratio (ESR) in rheumatoid arthritis patients [18]. In a clinical trial of rheumatoid arthritis patients, supplementation of 4.5 g L-His for 30 days showed clinical benefits in patients with more active and prolonged disease [20]. In chronic kidney disease, plasma His concentrations are inversely correlated with CRP and hepatocyte growth factor, markers that reflect inflammation [19]. Furthermore, enteral nutrition therapy using an elemental diet, with a His density more than twice that of the recommended WHO/FAO/UN requirement, has shown efficacy in CD [21]. Importantly, we previously reported that orally administered His ameliorates intestinal inflammation in an IL-10-deficient transfer mouse colitis model by inhibiting the production of pro-inflammatory cytokines by activated macrophages through intracellular mechanisms [22].

Plasma AA profiles are affected by the levels of protein and AA intake. Although the effects of the final meal on fasting concentrations of plasma AA are minimal, animal studies have shown that chronic alteration of protein intake affects fasting concentrations [10]. Therefore, the observed aminogram changes in IBD patients in the present study could be partially attributed to changes in dietary intake. Indeed, it has been reported that up to 85% of patients hospitalized with exacerbations of IBD have protein-energy malnutrition [23].

To date, several clinical markers that could aid the diagnosis of IBD and monitor disease activity have been reported. These include serological markers such as anti-neutrophil cytoplasmic antibodies (ANCAs) for UC and anti-*Saccharomyces cerevisiae* antibodies (ASCAs) for CD [24], [25], [26], although their practicality for clinical use remains controversial because of their inconvenience. Moreover, they do not reflect disease activity, so have limited use in monitoring the clinical course and therapeutic efficacy. Alternative candidate clinical markers capable of detecting inflammation and monitoring disease activity are fecal markers and serum CRP.

Fecal markers such as calprotectin have been shown to discriminate between active and inactive IBD, as well as non-IBD, based on intestinal inflammation [27]. However, although fecal markers may have a high sensitivity, their disease specificity is uncertain. Serum CRP is another non-invasive biomarker used to detect inflammation and monitor disease activity, especially in CD [27], [28], but its diagnostic accuracy remains controversial. By contrast, the MIAI is a convenient, non-invasive marker that may reflect disease activity more sensitively and specifically than other reported markers. Although the cost of plasma AA profiling in practical use is relatively more expensive than that of fecal calprotectin or serum CRP at present, we believe that it is possible to reduce the cost for testing plasma AA profiles by targeting several promising AAs and developing a novel enzymatic detection system of each AA as has been already achieved measurement of a BCAAs/tyrosine ratio (BTR). Since the MIAI integrates disease-specific alterations of AA metabolism, it can reflect individual conditions more objectively than analyses by solo factors (such as His). Although further investigations including prospective observations are required, the MIAI appears to be a promising marker that enables us to predict disease prognosis and manage patients accordingly.

In conclusion, the MIAI based on statistical modeling of plasma aminograms is a novel, non-invasive, objective parameter for the diagnosis and assessment of disease activity in IBD patients. It was shown to be capable of significantly discriminating between HCs and CD or UC patients with very high sensitivity and specificity, as well as between CD and UC patients, and those with active disease or undergoing remission. The introduction of metabolomics into IBD clinics has provided us with new insights and will contribute to the establishment of clinical parameters and our understanding of IBD pathophysiology.

## Supporting Information

Figure S1 **Scatter plots of plasma AA concentrations in individual HCs and IBD patients.** Each panel represents the scatterplot of two plasma AAs from individual HCs (green), CD patients (red), and UC patients (blue). Panel shows correlation plots of the concentrations of 22 AAs.
(TIF)

Figure S2 **A general imbalance of branched-chain AAs (BCAAs) in IBD patients.** (**A**) Correlation plots of leucine (Leu), valine (Val), and isoleucine (Ile). Ellipses show that 95% of the values are in a bivariate normal distribution. (**B**) Mean plasma Leu, Ile, and Val concentrations in active CD (CDa), remission CD (CDr), active UC (UCa), and remission UC (UCr) patients. Error bars show standard deviations. The two-tailed p-values are based on the Mann-Whitney U test.
(TIF)

Table S1 **Candidates of MIAI for clinical diagnosis of IBD.**
(DOC)

Methods S1 **Statistical analysis.**
(DOC)
