# Supplementary figures and images for: Novel, Objective, Multivariate Biomarkers Composed of Plasma Amino Acid Profiles for the Diagnosis and Assessment of Inflammatory Bowel Disease
Source: PLoS One. 2012 Jan 31;7(1):e31131. doi: 10.1371/journal.pone.0031131 (PMC3269436; doi:10.1371/journal.pone.0031131)

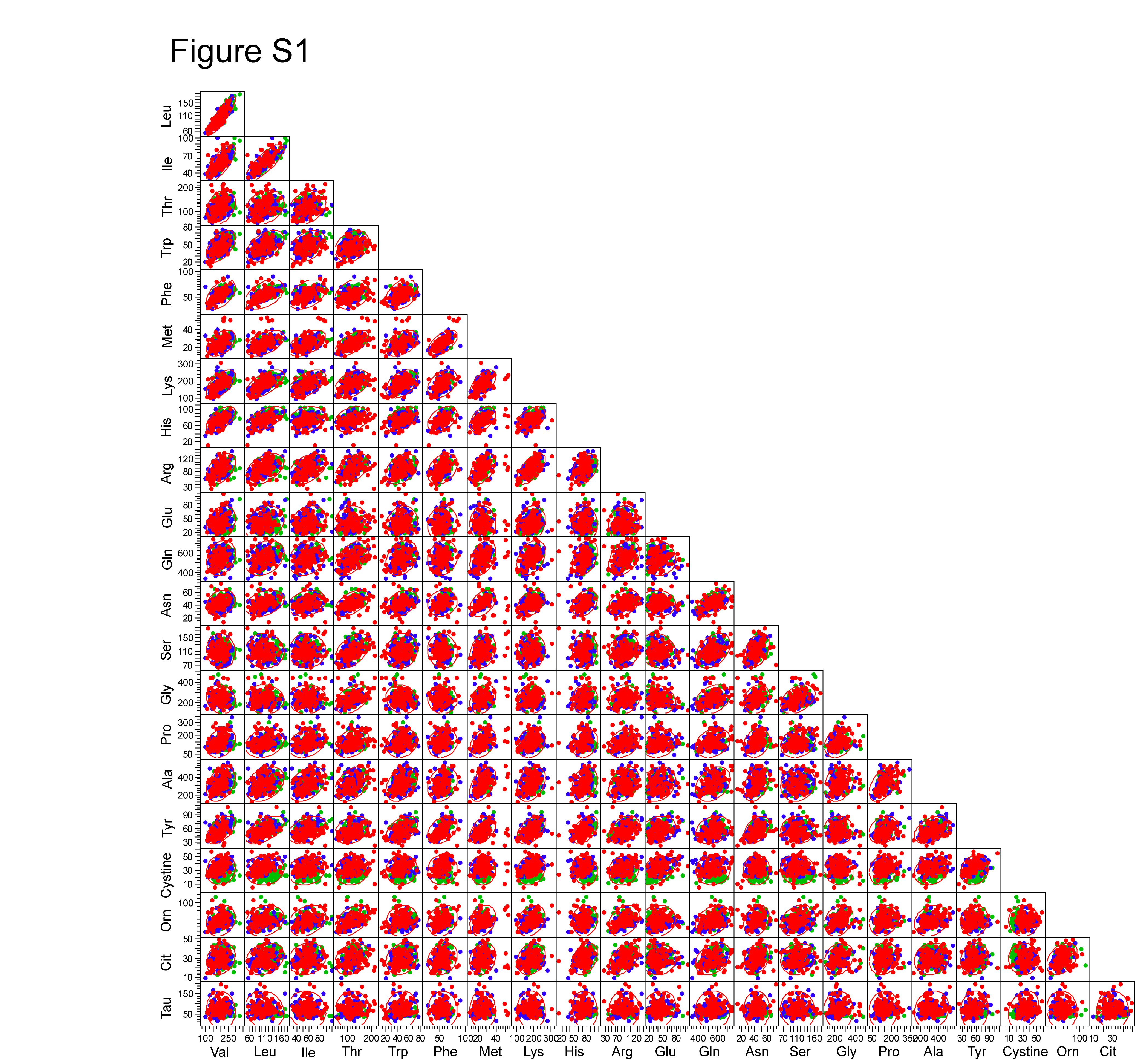

Supplement: Figure S1 — Scatter plots of plasma AA concentrations in individual HCs and IBD patients. Each panel represents the scatterplot of two plasma AAs from individual HCs (green), CD patients (red), and UC patients (blue). Panel shows correlation plots of the concentrations of 22 AAs. (TIF) [file pone.0031131.s001.tif]

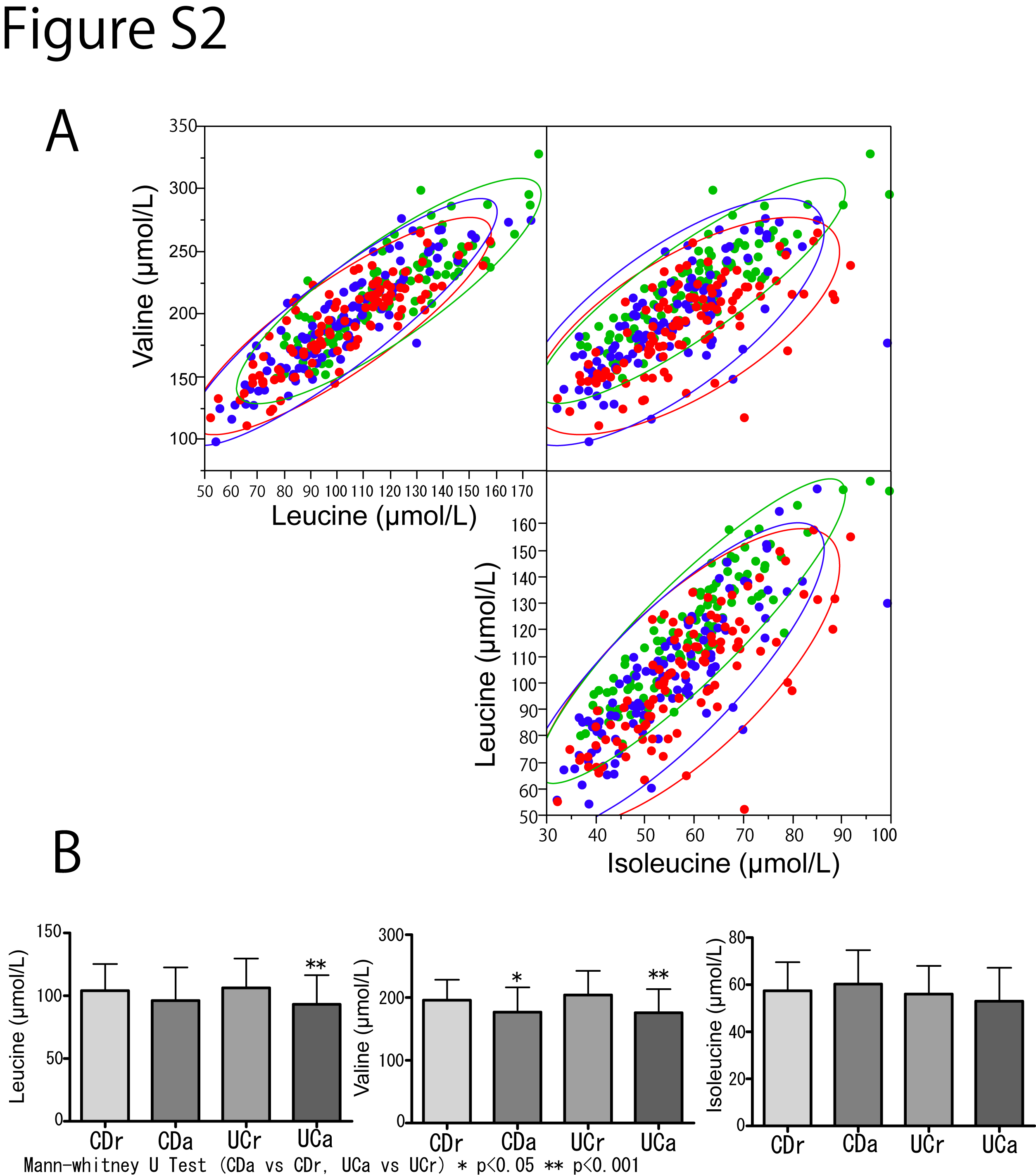

Supplement: Figure S2 — A general imbalance of branched-chain AAs (BCAAs) in IBD patients. (A) Correlation plots of leucine (Leu), valine (Val), and isoleucine (Ile). Ellipses show that 95% of the values are in a bivariate normal distribution. (B) Mean plasma Leu, Ile, and Val concentrations in active CD (CDa), remission CD (CDr), active UC (UCa), and remission UC (UCr) patients. Error bars show standard deviations. The two-tailed p-values are based on the Mann-Whitney U test. (TIF) [file pone.0031131.s002.tif]
