# Supplementary material for: Novel, Objective, Multivariate Biomarkers Composed of Plasma Amino Acid Profiles for the Diagnosis and Assessment of Inflammatory Bowel Disease
Source: PLoS One. 2012 Jan 31;7(1):e31131. doi: 10.1371/journal.pone.0031131 (PMC3269436; doi:10.1371/journal.pone.0031131)
Supplement: Table S1 — Candidates of MIAI for clinical diagnosis of IBD. (DOC) [file pone.0031131.s003.doc]

**Table S1. Candidates of MIAI for clinical diagnosis of IBD.**

CD vs HC

| No. | Index Candidate | Discovery Set | Validation Set |
| --- | --- | --- | --- |
| 1 | 22.4350+5.1842*[Tau]-2.6776*[His]-4.5197*[Tyr]-8.1647*[Val]+7.2102*[Ile] | 0.955 | 0.940 |
| 2 | 16.7041+5.0505*[Tau]-4.2026*[Tyr]-7.8409*[Val]-4.0529*[Leu]+9.8432*[Ile] | 0.953 | 0.944 |
| 3 | 13.2999+4.5998*[Tau]-3.7555*[His]-10.1206*[Val]+2.6630*[Pro]+6.0612*[Ile] | 0.952 | 0.957 |
| 4 | 13.2811+5.1982*[Tau]-5.3238*[Tyr]-10.9841*[Val]+2.9016*[Pro]+7.5706*[Ile] | 0.951 | 0.952 |
| 5 | 11.2366+4.7913*[Tau]-2.9082*[His]-4.6380*[Tyr]-5.4077*[Leu]+6.3011*[Ile] | 0.950 | 0.922 |
| 6 | 21.3188+4.6930*[Tau]-6.3291*[His]-5.5959*[Tyr]+3.7189*[Pro]-1.9190*[Ser] | 0.951 | 0.934 |
| 7 | 16.5403+4.7563*[Tau]-5.4126*[His]-5.2480*[Tyr]-1.7781*[Val]+3.6269*[Pro] | 0.950 | 0.927 |
| 8 | 10.8507+4.7316*[Tau]-6.6669*[His]-6.2591*[Tyr]+1.9764*[Arg]+2.5793*[Ala] | 0.949 | 0.911 |
| 9 | 14.4855+4.7999*[Tau]-6.0893*[His]-5.1686*[Tyr]+3.5622*[Pro]-1.2280*[Asn] | 0.950 | 0.935 |
| 10 | 17.5179+4.6221*[Tau]-3.5796*[His]-10.4456*[Val]+1.4878*[Arg]+6.8194*[Ile] | 0.950 | 0.941 |

UC vs HC

| No. | Index Candidate | Discovery Set | Validation　Set |
| --- | --- | --- | --- |
| 1 | 27.4215-7.5988*[His]+4.6212*[Tau]-2.1065*[Tyr]-4.9636*[Asn]+2.9296*[Thr] | 0.912 | 0.894 |
| 2 | 26.6682-7.5747*[His]+4.5718*[Tau]-2.5711*[Tyr]-3.8223*[Asn]+2.7603*[Arg] | 0.910 | 0.887 |
| 3 | 29.5836-7.7492*[His]+4.5235*[Tau]-5.6087*[Asn]-1.3604*[Val]+2.9366*[Thr] | 0.907 | 0.892 |
| 4 | 24.5431-8.8545*[His]+4.4941*[Tau]-5.6007*[Asn]+2.3236*[Thr]+1.1596*[Pro] | 0.907 | 0.896 |
| 5 | 34.3223-7.9823*[His]+4.4528*[Tau]-3.7864*[Tyr]-2.3816*[Gly]+2.3243*[Arg] | 0.907 | 0.883 |
| 6 | 23.2807-6.9235*[His]+4.8222*[Tau]-4.0988*[Tyr]-2.9801*[Cit]+3.0664*[Arg] | 0.910 | 0.879 |
| 7 | 35.2533-7.6755*[His]+4.4085*[Tau]-3.6309*[Tyr]-2.4975*[Gly]+1.7129*[Pro] | 0.905 | 0.899 |
| 8 | 26.9721-7.2217*[His]+4.5266*[Tau]-2.3159*[Tyr]-3.3649*[Asn]+1.6360*[Pro] | 0.906 | 0.901 |
| 9 | 23.1714-6.9546*[His]+4.4474*[Tau]-2.9450*[Tyr]-3.5163*[Asn]+2.4344*[Ala] | 0.908 | 0.887 |
| 10 | 31.1961-7.4221*[His]+4.3368*[Tau]-4.1913*[Tyr]-2.4143*[Gly]+2.3232*[Ala] | 0.906 | 0.882 |
