# Supplementary material for: Novel, Objective, Multivariate Biomarkers Composed of Plasma Amino Acid Profiles for the Diagnosis and Assessment of Inflammatory Bowel Disease
Source: PLoS One. 2012 Jan 31;7(1):e31131. doi: 10.1371/journal.pone.0031131 (PMC3269436; doi:10.1371/journal.pone.0031131)
Supplement: Methods S1 — Statistical analysis. (DOC) [file pone.0031131.s004.doc]

**Methods S1**

***Statistical analysis***

In multiple discriminant analysis, a logarithmic transformation was performed to normalize the distribution of plasma amino acid concentrations. Training models were developed using the Fisher linear classifier method^1^ in the discovery set. For each comparison analysis, the models were validated using a 10-fold cross-validation approach based on ROC AUC criteria. Briefly, the 10-fold cross-validation approach divided the randomized dataset into 10 datasets. The model was trained on nine of these subsets and then tested on the remaining subset. This procedure was repeated 1,000 times and the mean ROC AUC was estimated. The models for CD vs. HC, UC vs. HC, active CD (CDa) vs. active UC (UCa), CDa vs. remission CD (CDr) and UCa vs. remission UC (UCr) were computed and validated by a 10-fold cross-validation approach. We assessed the training models for UC vs. HC and CD vs. HC in the validation set.

**Reference**

[1] Anderson, T.W., 1996. R.A. Fisher and multivariate analysis. Statistical Science 11 (1), 20-34.
